# County Provision for the Insane

**Published:** 1885-11

**Authors:** James G. Kiernan

**Affiliations:** Central Music Hall; Chicago


### COUNTY PROVISION FOR THE INSANE.

*By* JAMES G. KIERNAN, M. D., *Chicago.*

(Read before the Chicago Medical Society, 19th October, 1885.)

Dr. Chas. H. Hughes says in a recent contribution to the subject of State provision for the insane: "We live in an age when every uttered sentiment of charity toward the insane is applauded to its remotest echo; an age in which the chains and locks and bars and dismal dungeon cells and flagellations and manifold tortures of the less humane and less enlightened past are justly abhorrent; an age which measures its magnificent philanthropy by munificent millions, bestowed without stint upon monumental mansions for the indwelling of the most pitiable and afflicted of the children of men, safe from the pitiless storms of adverse environment without, which are so harshly violent to the morbidly sensitive and unstable insane mind; an age in which he who strikes a needless shackle from human form or heart, or removes a cause of human torture, psychical or physical, is regarded as a greater moral hero than he who, by storm or strategy of war, taketh a resisting fortress; an age when the Chiarugi's and Pinel's, the Conally's and Tuke's of not remotely past history, and the Florence Nightingale's and Dorothea Dix's of our own time, are enshrined in the hearts of a philanthropic world with greater than monumental memory."

Bright as is the picture here portrayed, its bright tints are more than equaled by the shadows which come into bold relief, when the problem which is to be discussed to-night is studied. The insane in the majority of county institutions continue in a condition only paralleled by the condition of the insane in hospitals before the days of Pinel and Chiarugi. This may seem an extremely invidious declaration to make, but its truth is demonstrable by the perusal of the official documents of most of the states. In Illinois, for example, a State second to none in its humanitarian aims, which takes a prominent rank when provision for the insane is mentioned, the State Board of Charities writes as follows:

"The objection to the care of the insane by counties is that county boards will not ordinarily furnish, upon the county farm or even in county lunatic asylums, the necessary attendance and medical care. * * * It is not at all an uncommon occurrence for a county board to let the care of the paupers on the county farm to the lowest bidder. Even where an alms-housekeeper is selected on account of his supposed qualification for the position, because he is an efficient farmer and a kind-hearted man and his wife is a stirring housekeeper, it does not follow that either he or his wife have any special qualifications for the care of insane patients. The presence of such patients on the farm is felt by them to be a perpetual annoyance and peril, and they rid themselves of the bother of it in the easiest possible way by shutting up every lunatic, male or female, who gives them any trouble, in a separate room, and leaving him there without recreation, companionship, or employment. He is fed through a hole in the door. Sometimes he has a bed and sometimes he sleeps on straw; sometimes the room he sleeps in is warmed in winter, sometimes not. * * * He strips himself of clothing; daubs the wall with filth; for amusement he resorts to the vilest practices, and thus he vegetates until he rots. This, of course, is a description of what occurs in extreme cases, but quite often. * * * The condition of the insane in these establishments is deplorable. Kindly treated by some keepers, by others they are regarded and treated as if they were animals, not men—indeed, not so well as animals capable of earning money for their owners

They are neglected, abused, confined with chains, locked up, left in nakedness and filth, caged, and not a soul has for them a friendly word. The medical supervision of them is totally inadequate; they have no proper personal attendance; they are without amusement or occupation of any sort. Some of them are taken out at long intervals for an airing or to be washed, possibly by standing them naked in a corner and throwing water upon them with a hose. Others remain in their cells or dens from one end of the year to the other. Their mental malady is aggravated by neglect and cruelty and their only hope in death." Such is the language of the Illinois State Board of Charities, late in the nineteenth century, respecting the county provision for the insane in this State, and these statements supported by facts, for example, in Adams county: "It is cruel and improper to confine thirty-two insane in their rooms without opportunity for freedom of action or recreation. A man spoken of as under restraint, wears a chain. A female insane pauper, who put out her eyes, occupies a large wooden box filled with straw in company with another blind insane woman. They are both filthy in their habits, and neither of them wears any clothing." Brown county: "An insane man is very filthy in his habits and is kept at night in an out-house, where he sleeps on straw in a box on the floor. The straw is not renewed as often as it should be." Champaign: "The condition of the insane department is very bad. The rooms are filthy, the beds and bedding ragged and dirty." De-Kalb: "One insane man is locked up in his cell and is not taken out for any purpose. He is filthy in his person, and his room is in the same condition." DeWitt: "There is an insane pauper on this farm who wears a ball and chain." Fulton: "The insane department is entirely unfit for use and should be abandoned. The ventilation is poor and a foul odor pervades it throughout. A insane man is nude all the time and is rarely if ever taken from his cell. * * * An insane woman is also nude most of the time; both of them filthy in their habits and exposed freely to the gaze of every inmate on the farm who chooses to look at them. * * * The sexes are not separated." Hancock: "A large number of the insane are imprisoned in their rooms from one end of the week to another." Kankakee: "Some of the insane go naked at times." Lake: "The insane locked in their cells in the basement are much to be pitied. Four of these cells were found to be very filthy and the odor from them was very offensive. One insane man is very violent. His entire costume is a shirt and handcuffs. The handcuffs were bright; the shirt was not." McDonough: "One of the insane men wears a chain." Mercer: "One insane woman is sometimes locked in a cell as dark as a dungeon." Moultrie: "An insane woman occupies a pen in the winter time, which is placed in the corner of a room. The dirty condition of the premises is a disgrace to the keeper." Peoria: "Three insane men are confined in cages in the basement. These cages are in very bad order and very offensive on account of the filty habits of the inmates, who are naked a great part of the time." Union: "An insane woman spends most of her time in a sitting position with her knees drawn up to her chin. She destroys bedding and clothing and sleeps in a pen in one corner of a room. The pen is three feet wide by six long, and the bedding consists of some straw and old tattered quilts, which were in a filthy condition." Whiteside: "It has beautiful grounds in front. Its weak point is the insane department. Of the forty inmates, twenty were insane and sixteen in seclusion. It is impossible that any necessity exists for secluding so large a proportion of the insane." They "do not receive a sufficient amount of food."

These citations at random from recent reports of the State Board of Charities illustrate that the insane in the county almshouses are in a pitiable state. The history of the insane in Cook county has passed through much the same phases.

"Without a very efficient superintendence, chiefly to be exercised by the chief medical officer, the mere absence of mechanical restraint may constitute no sufficient security against neglect or even ill-treatment of patients in a large asylum. The medical officers who consider such watchful supervision not properly comprised in their duties have formed but a very inadequate conception of them."

In consequence of the public outcry, a seemingly complete change in the management of the institution was made. A new superintendent was appointed, the wardenship was abolished, and a consulting board composed of three reputable neurologists, Drs. Jewell, Lyman and Brower, and a medical politician, was appointed. In consequence of the repeated pigeonholing of his suggestions and the complete ignoring of them by the Charities Committee, which continued to run the institution on a political basis, Dr. Jewell resigned, and a year later, in consequence of the intrigues by the medical politician and the medical superintendent, Drs. Lyman and Brower also tendered their resignations. From this time on the institution was run on a political system, but of its inside history little is obtainable until the year 1883, when Dr. S. V. Clevenger was elected special pathologist.

Through his suggestions, which stimulated outside influences, a lady physician was appointed. This lady had been a missionary in China, given to self-sacrifice; a lady of rare personal merit, ability and conscientiousness. Of the period 1883–4 she writes from her personal observation: "From the first I was struck by the lack of system or organization that prevailed.

No histories of cases by the physician in charge were kept; no census, and very meagre records of any description. The visits of the superintendent to the wards were few and hasty. In each ward was kept a bottle of whisky and a bottle of strong sleeping medicine of bromides and chloral which the attendants dealt out at their discretion. Many times on being called to a patient I received this history of the case: 'I gave her a drink of whisky and then a dose of sleeping medicine, but she did not get any better, so I called you.' It took some time to impress the idea that I preferred to be called before the ever-ready remedies were used. Evidently a physician had been a luxury and only called as a last resort. I have known of attendants hiring patients to work for them by giving them whisky and sleeping medicine, which they (the patients) had come to crave as the opium-eaters their opium. The amount of this sleeping medicine used in the female wards alone was enormous, as was also the whisky. It is safe to say the amount used in the female wards alone, with less than 300 patients, was twenty times more than is used in the entire institution of over 1,400 patients at Kankakee, and the noise at the latter institution is much less than at the Cook County Asylum. That the attendants, both male and female, helped themselves quite largely from the ward whisky-bottle, which was filled whenever they desired, is beyond doubt. The real needs of the patients seemed to call for no thought. They had no bath-towels, and attendants were in the habit of putting the clothing on the patients without drying the skin. The wards were frequently cold, and the patients had no winter clothing. Many who would have been benfited by out-door exercise did not leave the ward once for six months, because there were no wraps. No system was adopted in regard to clothing, and no account taken of what patients brought to the hospital. The bedding was at all times insufficient. Restraint was used at the discretion of the attendant, and I have seen a patient jacketed, unable to use her hands, eat her food from the platter like a wild animal. The food is almost beyond description. Where in the State institutions will you find deaths from scurvy frequent? Where but at the Cook County Asylum will you find two patients fiercely fighting for a small potato, given only as a Sunday luxury? Where will you find the hog's head, hair and all, given to the patients? I have often picked out the half of a hog's ear, with the hair on it, from a dish set before the patient to eat. I have picked out bunches of hair half as large as my little finger from the patient's food. Dying patients, if fed at all, were fed on sour milk. The milk, which is so great a necessity in the treatment of the insane, was almost never fit for use. They had meat never more than once a day, and often not that. The scurvy alone will speak for the vegetables. Whisky and sleeping medicine seemed to be the only articles of diet which never ailed. The drug-room was the greater part of the time turned into a saloon. Often I have had to wait for a prescription which was needed for an urgent case, until the druggist had served with beer, port, sherry or whisky, a room full of men. I never visited the drug store but with trepidation, and always breathed more freely when I left its degrading atmosphere." Of the same period, a lady attendant writes:

"As I was attendant in two State institutions prior to entering Jefferson, I was in some measure prepared to form an opinion of the management of the institution during 1883-84. Management or system there was none. The attendants on the female side of the house indulged freely in stimulants, and I have on more than one occasion observed at least three of them under the influence of liquor. Some of them used, in presence of the male attendants, decidedly coarse language.

In every ward a bottle of sedative mixture and a bottle of whisky were kept, and these were administered freely by the attendants. It was a common remark: 'It is no use doing anything for these cranks.' The physician was called only as a last resort, and though diarrhœa and scurvy were very frequent, but little attention was paid to the diet; sick patients were fed with the same food as the others. The great article of diet was pigs' heads, boiled without being shaved or cleaned. The meat frequently stank. The clothing and cleanliness of the patients received but little attention. For weeks and weeks we were without fine-tooth combs. There was no discipline. The engineers and other mechanics had keys to the female wards, and the assistant engineer frequently visited them in an intoxicated condition, often cursing the attendants. Every Saturday a dance was held, and beer kegs were frequently brought up into the dance hall and emptied by the attendants and employés after the patients had retired, festivities being kept up till morning. Restraint was used by the attendant at discretion. The night watch paid but little attention to their duties — how little may be seen from the fact that a patient was found dead one morning, partially eaten by rats. The superintendent never went through the wards except in company with visitors, but every morning stuck his head in the door and asked if all was right. The washing of the patients frequently disappeared when sent to the laundry. I saw what I never saw in any institution, the superintendent douse with cold water a patient in wristlets because she called him names. This was during the superintendency of Dr. Spray. I was asked if I would take the position by a medical friend and accepted, not knowing until too late that a warden was to be elected who had been a keeper of a saloon in which gambling was practiced, nor until much later that he was to be supreme. How the incongruity of such procedure of the Board struck intelligent laymen may be gleaned from the newspaper reports of the time, of which I cite one:

"'After going about the grounds the committee met in the parlor of the asylum for the purpose of deciding upon what the duties of the Warden and Medical Superintendent should be. No two Commissioners could agree, and after the committee had adjourned it left the reporters present in doubt as to whether the head cook or the chief doctor was the real manager of the institution. There was a wrangle as to whether the new Medical Superintendent or the Warden should be the head of the establishment. It was finally decided that the Medical Superintendent should be held responsible for the hiring and discharging of the nurses, and that the Warden should be responsible for the general management of the institution outside the medical department. The Medical Superintendent is supposed to be responsible for the inmates, but it is left with the Warden to hire the supervisors of the wards, whose duty it is to watch the attendants of the lunatics and also to dispense the medicines to the patients. Chairman McCarthy was appealed to to help the committee out of their muddle, and, as he had helped to put the management of the institution in its present fix, the only thing he could recommend was to have the Warden make all appointments of attendants in the asylum, and at the same time hold the Medical Superintendent responsible for the latter's actions. The wisdom of this suggestion was so obvious that Commissioner Ochs said it was a piece of d——d nonsense to let the Warden hire the supervisors of the different wards and at the same time hold the Medical Superintendent responsible for their actions. This objection was clearly well taken, but the Commissioners not being able to meet it passed it after their usual careless manner and hurriedly adjourned their session, leaving the Medical Superintendent and the Warden of the establishment to decide the question of supremacy in office between themselves. Dr. James G. Kiernan is undoubtedly an able physician, but should Varnell, the newly-appointed Warden, and himself get at loggerheads as to which of the two is in authority, the matter would give the County Board something to think over.'

"Within three months all power was taken from me except the right to enter protest. I found the institution in a wretched condition. There was no means of determining just what patients were in the building, and, as a careful census revealed, there were four more patients on the register than in the institution. In a remote ward patients were found with ulcers swarming with maggots, and none of the patients of the ward had been out for months. The drug store was destitute of the most necessary articles, there being but one drachm of quinine in the whole building, and many patients ill from low types of intermittent fever. The drug store was a gin mill to which employés invited the politicians who visited them. The druggist, a conscientious, able pharmacist, complained bitterly of being turned into a bar-tender, as he did also of the vile drugs sent him. The bread was badly baked and full of lumps, and the baker was half drunk most of the time, so that I was able to soon secure his dismissal. The male attendants were, as a rule, coarse, brutal men, and within two months I had occasion to discharge five of them for striking patients; receiving my first taste of the discipline hitherto prevalent in the institution by being knocked down and called a "crank" for interfering with one individual amusing himself by pounding a dement. The Saturday night dances already mentioned were marked by the conduct described ten years ago by the *Tribune*

article. The female wards were visited at all hours by the male employés, most of whom had keys. I issued a few necessary orders and made an attempt to regulate these and other evils in the first month, and a great turmoil followed. Of some of this turmoil Dr. Clevenger gives a good description.

"DR. KIERNAN'S first order was for the attendants to restrain no patient without an order from a physician, which were all recorded in a restraint book. A great uproar followed. This was an unheard-of proceeding there, and much nonsense was uttered over the new order. The next order was that the night-watch should not issue medicine to the patients at their own will, but were to call upon the physician. The watchmen conspired to make the physicians sick of tho arrangement by waking them up repeatedly and unnecessarily, but the promptness of the doctors defeated the watchmen, and many of the patients rested well without sleeping medicine being given them. Among the new arrangements which disgusted the gang, was an order that all who were employed were required to take off their hats when going through the wards and to address the patients as Mr., Mrs. or Miss, as the case might be, in lieu of more familiar and contemptuous appellations hitherto commonly in use." How little was accomplished despite strenuous efforts may be gleaned from the following record, kept by DR. HOWE, Nov. 7, 1884: "The condition of the female patients as regards clothing was and is pitiable. In one ward there are thirteen beds which have no clothing, except a sheet and a thin spread. The other beds of the ward have but one thin spread or blanket in addition. This condition was reported six weeks ago, but nothing has been done to remedy it. In many of the wards there is not enough underclothing to go round, to say nothing of a change for each patient, and freequently when the wards have a temperature of 60° F. aged and feeble patients have but two garments to cover them. Patients whose recovery depends on out-door exercise cannot go out because they have nothing to keep them warm. Others, who have been ordered milk diet, could not get the milk on the doctor's order. While the patients have been suffering from lack of clothing, the housekeeper and other employés have been keeping many of them at work on quilts and fancy work for their own ease. The doctor, anxious to get the patients clothed, urged them instead of doing fancy work to put their time to the necessary sewing and knitting. The Medical Superintendent issued an order that the patients were to do no fancy work without his order. The housekeeper openly violated this rule, and repeatedly brought work surreptitiously to the patients, thus encouraging them in the belief that the Medical Superintendent was persecuting them by not allowing them to do fancy work. In one ward eight garments were made in one day by the patients, and the moral influence of the idea that they were working for each other was decidedly beneficial, and as much so as the hopeless, unending fancy work was detrimental. The warden forbade any cloth to go to the wards, effectually nipping in the bud the healthy enthusiasm which the doctor and some of the more interested attendants succeeded in arousing. Patients have been shut in their rooms and told they could not come out till a certain amount of fancy work was done for some employé. The housekeeper has come round the wards with her apron full of fruit and other things, and given them promiscuously around without reference to the physical condition of the patients. Troublesome cases of dyspepsia were fed on pie, diarrhœa patients on fruit. She never brought fruit into the wards until the order about fancy work was issued, and fruit asked for for a case of scurvy was denied contemptuously by her." As an illustration of how the temperature of the building was attended to, the following citation will serve: History Book D 2, November 7, 1884. Mary Ann D. "Has been worse since local treatment was stopped, which I was obliged to do on account of my room being cold, so that I had no suitable place for treatment." I pass without comment over the numerous assaults made on myself, and would only call attention to the facts that, during April, May and June, 1885, no efforts of mine could secure fine-tooth combs for the patients until I sent out to another institution and borrowed them, becoming personally responsible. During the same period the practice of allowing the mechanics (most of whom were ward workers and knew nothing about their trade, which they learned after appointment,) keys to the female wards continued, and one of these men was detected, somewhat *en dishabille*, attempting to enter a female ward at one o'clock at night. The keys were then taken away, but were given back after September, and on the night of September 9, a calciminer was detected attempting to enter the female ward. Of the food during this period I shall need to cite but one instance. Despite my efforts, no dietary table was ever adopted. In consequence of a suicide, due to neglect by an attendant whom I had long tried to have discharged, a species of examination of everything was made by the Charities' Committee. The cook was asked if the food was always good, and despite the fact that there were twenty cases of scurvy in the house that very day, said, Yes. Whereupon the lady physician held up the iron-ringed, unwashed snout of a pig suffering from catarrh, which had been taken from the patient's food that very day and asked if that were a specimen of the good food. The decision of the Commissioners was that the only way to prevent the finding of these things in the food was to have no lady physician, and her resignation was thereupon demanded.

I have detailed enough to show that for the past two years Cook County Hospital for the Insane differs from all other institutions of the kind in being a refuge for ward workers. And now comes the serious question of cost. I quote from the *Daily News*, having verified the figures from the New York State Commissioner on Lunacy's Report and that of the Illinois State Board of Charities:

"A comparison of the cost of maintaining the indigent insane will give some idea of the magnitude of the steal in this one line. Last year the 567 Cook County Insane Asylum patients cost $188,039, being at the rate of $331.63 each. During the same time 841 patients at Binghampton, N. Y., cost $72,055.52, 533 at Elgin cost $109,519.33, 595 at Anna cost $115,360.84, 633 at Jacksonville cost $112,888.45, 639 at Kankakee cost $101,232.80, 1,743 at Willard, N. Y., cost $273,949.48, and 1,285 in the New York County Insane Asylum cost $162,569.34. The average cost of each insane patient in the Illinois State institutions was $184.02, and in the New York institutions it was $184.23. In the New York County Insane Asylum it was $126.51." To these figures may be added some taken from Alabama.

COOK COUNTY.

| | Patients. | Cost. |
|---|---|---|
| 1883 | 597 | $170,000 |
| 1884 | 567 | 188,039 |
| | 1,164 | $358,039 |

ALABAMA.

| | | |
|---|---|---|
| 1883 | 438 | $63,834.89 |
| 1884 | 555 | 72,897.83 |
| | 993 | $136,732.72 |

When it is remembered that the Alabama and New York institutions are among the best managed institutions in the country, the contrast becomes startling. I say nothing of the discrepancy of $53,000 between the 1883 report of Superintendent Spray and the report of the Clerk of the Board; $117,000 being expended, according to Dr. Spray, and $170,000 according to the clerk of the Board. Having pointed out the evils, it now becomes necessary to suggest a remedy. The condition of the hospital is to day substantially what it was ten years ago, and a change in individuals works but little improvement.

A complete change in the system is required, and this can be secured only in one way, and that is by giving the State Board of Charities, as I suggested in this SOCIETY two years ago, and as is done in Wisconsin, complete control over all almshouse expenditures and appointments. All the evils mentioned result from deficient power of supervision and control by the State Board of Charities, and this Board should also be aided, as in New York, by volunteer visitors in the different counties, to whom they could delegate their powers. Laws to secure these results should be passed when the Legislature meets, for until this is done the condition of the insane in county institutions will remain, as it is, a disgrace to Illinois.

Central Music Hall.

